# Herpes Simplex Virus Type 1 Infection Induces the Formation of Tunneling Nanotubes

**DOI:** 10.3390/microorganisms11081916

**Published:** 2023-07-28

**Authors:** Jie Wang, Kun-Te Shang, Qiong-Hong Ma, Zhao-Ying Dong, Yi-Hong Chen, Yu-Feng Yao

**Affiliations:** 1 Department of Ophthalmology, Sir Run Run Shaw Hospital, Zhejiang University School of Medicine, 3 Qingchun East Road, Hangzhou 310016, China; wangjieliu@126.com (J.W.);; 2 Key Laboratory for Corneal Diseases Research of Zhejiang Province, 3 Qingchun East Road, Hangzhou 310016, China; 3 Department of Ophthalmology, The First Affiliated Hospital of Zhejiang Chinese Medical University (Zhejiang Provincial Hospital of Chinese Medicine), 54 Youdian Road, Hangzhou 310003, China; 4 Department of Ophthalmology, The Third Affiliated Hospital of Zhejiang Chinese Medical University, 219 Moganshan Road, Hangzhou 310005, China

**Keywords:** herpes simplex virus type 1, virus transmission, tunneling nanotubes

## Abstract

Herpes simplex virus type 1 (HSV-1) is human specific virus. The intercellular transmission of HSV-1 is essential in its pathogenesis. The tunneling nanotube (TNT), a new mode connecting distant cells, has been found to play an important role in the spread of various viruses like human immunodeficiency virus (HIV) and influenza virus. However, whether HSV-1 can be transmitted through TNTs has not been confirmed. The purpose of this study was to clarify this, and further to determine the effect of inhibiting the actin-related protein 2/3 (Arp2/3) complex on the intercellular transmission of HSV-1. A scanning electron microscope and fluorescence microscope detected the formation of TNTs between HSV-1 infected cells. Envelope glycoprotein D (gD) and envelope glycoprotein E (gE) of HSV-1 and viral particles were observed in TNTs. Treatment with CK666, an inhibitor of the Arp2/3 complex, reduced the number of TNTs by approximately 40–80%. At the same time, the DNA level of HSV-1 in cells and the number of plaque formation units (PFU) were also reduced by nearly 30%. These findings indicated that TNT contributes to HSV-1 transmission and that the inhibition of the Arp2/3 complex could impair HSV-1 transmission, which not only provides a novel insight into the transmission mode of HSV-1, but also a putative new antiviral target.

## 1. Introduction

Herpes simplex virus type 1 (HSV-1), a neurotropic alphaherpesvirus specific to human beings, is generally susceptible in the human population. Although the occurrence of HSV-1 has decreased in some regions, the prevalence rate worldwide remains high [1]. In 2016, about 3752 million people were infected with HSV-1 [2]. HSV-1 usually infects epithelial cells such as the cornea or oral mucosa. After replication, new progeny HSV-1 disseminate to neighboring cells. The virus is then carried via retrograde axonal transport to the sensory ganglia, usually the trigeminal ganglion, establishing lifelong latent infection and the periodic reactivation of infected sites [3].

The intercellular transmission of HSV-1 plays a key role in the infection, latency and recurrence of ocular and orolabial herpes. The tunneling nanotube (TNT) is a long-distance intercellular nanotubes connection. It was first described in the ultrafine intercellular structures of PC12 cells in 2004, and was speculated to partake in intercellular transport [4]. In 2008, it was found for the first time that a virus (HIV-1) can be transmitted through TNT [5]. Subsequent studies found that viruses such as orthomyxoviruses (e.g., influenza A virus, IAV) [6] and alphaherpesvirus (e.g., pseudorabies virus, PRV) [7] can spread through TNTs. The formation of TNT is a new mode of virus transmission, which facilitates the virus to spread to non-adjacent cells. However, the role of TNT in HSV-1 transmission has rarely been reported. 

Although TNT has been found to participate in virus transmission, the exact mechanism of TNT formation, and the impact of inhibiting TNT formation on virus transmission, remains unclear. Some studies have shown that TNT formation is based on filamentous (F)-actin [4,8,9], as the polymerization of F-actin triggers the initiation of TNT protrusion [10,11]. In addition, F-actin arises from the branched actin network generated by the actin related protein 2/3 (Arp2/3) complex, which is crucial in mediating the formation of branched actin filaments and assisting in the assembly of F-actin. The inhibition of Arp2/3 complex activation affects the polymerization of F-actin [12,13,14]. However, the impact of inhibiting Arp2/3 complex activation on TNT formation and HSV-1 transmission is still unclear.

In order to examine whether HSV-1 can trigger the formation of TNTs, and whether TNTs contribute to the intercellular spread of HSV-1, we observed the connections between intercellular spread and the transmission of HSV-1 between non-adjacent human corneal epithelial cells (HCECs), and between Vero cells. Furthermore, we also investigated the effects of pharmacological inhibitors of Arp2/3 complex (CK666) on the formation of TNTs, and on the efficiency of HSV-1 spread. We confirmed that HSV-1 can induce the formation of TNTs and spread via TNTs, and that CK666 plays an important role in reducing the formation of TNTs and weakening the spread of HSV-1.

## 2. Materials and Methods

### 2.1. Cell Culture

Human corneal epithelial cells (HCECs) (Gift from the Riken Institute of Physical and Chemical Research, Tokyo, Japan), as previously described [15], were cultured in Dulbecco’s modified Eagle’s/F12 medium (DEME/F12) (Zhejiang Senrui Biotechnology Co., Ltd., Huzhou, China) supplemented with 10% fetal bovine serum (FBS) (Gibco, Thermo Fisher Scientific, Waltham, MA, USA) and 1% penicillin-streptomycin-glutamine (Gibco, Thermo Fisher Scientific, Waltham, MA, USA). African green money kidney cells (Vero cells) (ATCC, CCL-81, Manassas, VA, USA) were cultured in Dulbecco’s modified Eagle’s medium (DMEM) (Zhejiang Senrui Biotechnology Co., Ltd., Huzhou, China) supplemented with 5% FBS and 1% penicillin-streptomycin at 37 °C in 5% CO_2_. All cells were free of mycoplasma. Cell culture on three-dimensional gel was conducted as follows: add type I collagen (1 mg/mL) (Beijing Solarbio Science Technology Co., Ltd., Beijing, China) to the 24-well plate at room temperature, after the gel is solidified, seed 1 × 10^5^ HCECs with 500 μL medium on the gel, and then transfer them to the incubator at 37 °C and 5% CO_2_. 

### 2.2. Virus

Wild-type (WT) HSV-1 strain KOS (ATCC VR-1493) was stored at −80 °C. Virus replication and growth in HCECs and Vero cells were analyzed with plaque assays (as explained later, in the Plaque Assays section). The cells were seeded with 1 × 10^5^ in a 24-well plate with 500 μL medium, and subconfluent cells were infected at multiplicity of infection (MOI) of 1. Then, the cells were washed three times with phosphate-buffered saline (PBS) and cultured with complete medium for another 24 h, and finally collected and fixed at 24 h post infection (h.p.i.) for subsequent tests. 

### 2.3. MTT Assay

The 10^4^ HCECs and Vero cells in 96-wells plate were treated with various concentrations of CK666 (AdooQ Bioscience LLC., Irvine, CA, USA) (0, 0.1, 1, 5, 10, 20, 50, 100 μM) and 0.1% dimethyl sulfoxide (DMSO, as a drugs vehicle) (Sigma, Cream Ridge, NJ, USA) for 24 h. After incubation with CK666 and DMSO, cells were incubated with 5 mg/mL concentration of MTT (3-(4,5-Dimethylthiazol-2-yl)-2,5-diphenyltetrazolium bromide) (Beyotime Biotechnology Co., Ltd., Haimen, China) reagent in media for 4 h. Then, cells were incubated with Formazan solvent for 4 h and absorbance recorded using Multiskan Spectrum at 570 nm (Multiskan GO, Thermo Scientific, Waltham, MA, USA). Three replicates were performed. 

### 2.4. Drug Treatment

To observe the role of CK666 in inhibiting actin polymerization in HCECs and Vero cells, HCECs and Vero cells were infected with HSV-1 KOS virus for 1 h, then cells were incubated in medium containing either 50 μM CK666 or 0.01% DMSO (carrier) for 24 h. Changes in cells were observed with fluorescence microscopy imaging and plaque assays.

### 2.5. Immunofluorescence Staining

The HCECs and Vero cells were fixed with 4% paraformaldehyde (PFA) and permeabilized with 0.5% Triton X-100 (Beyotime Biotechnology Co., Ltd., Haimen, China) for 15 min, then blocked with 10% bovine serum albumin (Sigma, St. Louis, MO, USA) at 37 °C for 1 h. After performing washes three times, the samples were incubated with rabbit anti-HSV-1 gD antibody and rabbit anti-HSV-1 gD antibody (diluted at 1:50, Beijing Bioss Biotechnology Co., Ltd., Beijing, China) for 1 h at 37 °C. After washing, samples were incubated with Alexa Fluor 647 goat anti-rabbit (Alexa Fluor 647) (Beyotime Biotechnology Co., Ltd., China) and Goat Anti-Rabbit IgG H&L (Alexa Fluor 555) (Beijing Bioss Biotechnology Co., Ltd., Beijing, China) secondary antibodies (diluted at 1:500) at 37 °C for 1 h. Then, the sample was incubated with Alexa Fluor 488 phalloidin-FITC (actin-Tracker Green) (diluted at 1:100, Beyotime Biotechnology Co., Ltd., China) at 37 °C for 1 h. Finally, the nuclei were stained with 50 mg/mL 4′,6-diamidino-2-phenylindole (DAPI) (Beyotime Biotechnology Co., Ltd., China). Cells were analyzed for actin organization using fluorescence microscopy (U-HGLGPS, Olympus Corporation, Tokyo, Japan). A total of 150~200 cells were observed for each experimental group, and data were shown of three replicates. 

### 2.6. TNT Counting

TNT formation was quantified after 24 h of HSV-1 infection with or without CK666 incubation. About 0.1 million cells were seeded per well of a 24-well plate. Cells were fixed and stained with Phalloidin to stain the actin filaments. In this study, TNTs were defined as thin membranous structures at least ≥5 µm in length. TNTs were counted in 150~200 cells, and the experiment was repeated three times.

### 2.7. Scanning Electron Microscopy (SEM)

Cells were fixed using 2.5% glutaradehyde for 2 h at room temperature. Next, the cells were washed three times with 0.1 M PBS, and then the sample was fixed with 1% osmic acid solution for 1.5 h. The osmic acid solution was removed and the cells washed three times with PBS. After washing, the cells were dehydrated using a graded series of 30%, 50%, 70%, 80%, 90% and 95% alcohol concentrations for 15 min and for 20 min in 2× absolute ethanol, followed by critical point drying with carbon dioxide. Samples were sputtered with a 10 nm gold/palladium layer and observed with NOVA NANOSEM 450 (FEI, Hillsboro, OR, USA) at a voltage of 5 kV. 

### 2.8. Transmission Electron Microscopy (TEM)

Cells were fixed using 2.5% glutaradehyde for 2 h at room temperature, washed three times with 0.1 M PBS, and a post-fixation step was performed by incubating cells with 1% osmium tetroxide solution for 1 h. After washing three times, the cells were stained with 2% uranyl acetate for 30 min and dehydrated using a graded series of 50%, 70%, 80% and 90% alcohol concentrations for 10 min, for 15 min in absolute ethanol, and for 15 min in 100% acetone. The sample was placed in absolute acetone and final Spurr resin (1:1) at room temperature for 2 h, then transferred to pure Spurr resin for 2 h at 37 °C. Ultrathin sections were sliced on a Leica UC7 slicing machine and stained with uranyl acetate. Finally, the samples were observed with Tecnai G2 spirit (Thermo Fish Scientific Inc., Waltham, MA, USA) at a voltage of 120 kV.

### 2.9. Quantitative Polymerase Chain Reaction (qPCR)

The extraction of virus DNA was carried out using the PureLinkTM Viral RNA/DNA Mini Kit (Thermo Fish Scientific Inc., Waltham, MA, USA), according to the manufacturer’s instruction. Cells were lysed with proteinase K, lysis buffer and ethanol. Then, the DNA was purified by centrifugation. Finally, 40 μL sterile RNase-free water elution of DNA was used. A total of 20 μL of the qPCR reaction system was prepared with probe qPCR Master Mix (Takara, Shiga, Japan). The final concentration of forward and reverse primers was 200 nM. The PCR thermal cycling program is as follows: 95 °C for 10 s and 58 °C for 40 s with the reaction cycle of 45. Fluorescence signals were recorded with a Roche LightCycler480. The HSV-1 primers were Forward, CGTCCCTGTCCTTTTTCCCA; Reverse, ACGTAGCACGGTAGGTCAC; Probe, AAGCATCGACCGGTCCGCGCTAGTT. We used an HSV-DNA polymerase plasmid dilution (10^6^ copies, 10^5^ copies, 10^4^ copies, 10^3^ copies, 10^2^ copies and 10^1^ copies) to generate the standard curve. Gene expression was calculated using the standard curve and CT value. 

### 2.10. Plaque Assays

HCECs and Vero cells were infected with HSV-1 at an MOI of 1, and treated with or without 50 uM CK666. Then, the cell lysates of HCECs and Vero cells were collected and the confluent Vero cells infected. After adsorption in 5% CO_2_ at 37 °C for 1 h, the medium was removed and the cells washed with PBS three times, then medium added with 1.5% methylcellulose (Sigma, Cream Ridge, NJ, USA). At 72 h.p.i., cells were fixed with methanol-acetic acid (2:1) for 1 h, then stained with crystal violet and visualized using microscopy. The number of plaques was measured. Data shown represent the mean ± SD of triplicate assays. 

### 2.11. Statistical Analysis

Statistical analyses were performed using SPSS 18 (SPSS Inc., Chicago, IL, USA). Student’s *t*-test and ANOVA were used to determine the significance of differences between the groups. Statistical significance was defined as a *p* value less than 0.05.

## 3. Results 

### 3.1. Human Herpes Virus Type 1 (HSV-1) Induces the Formation of TNTs

**Figure 1:**
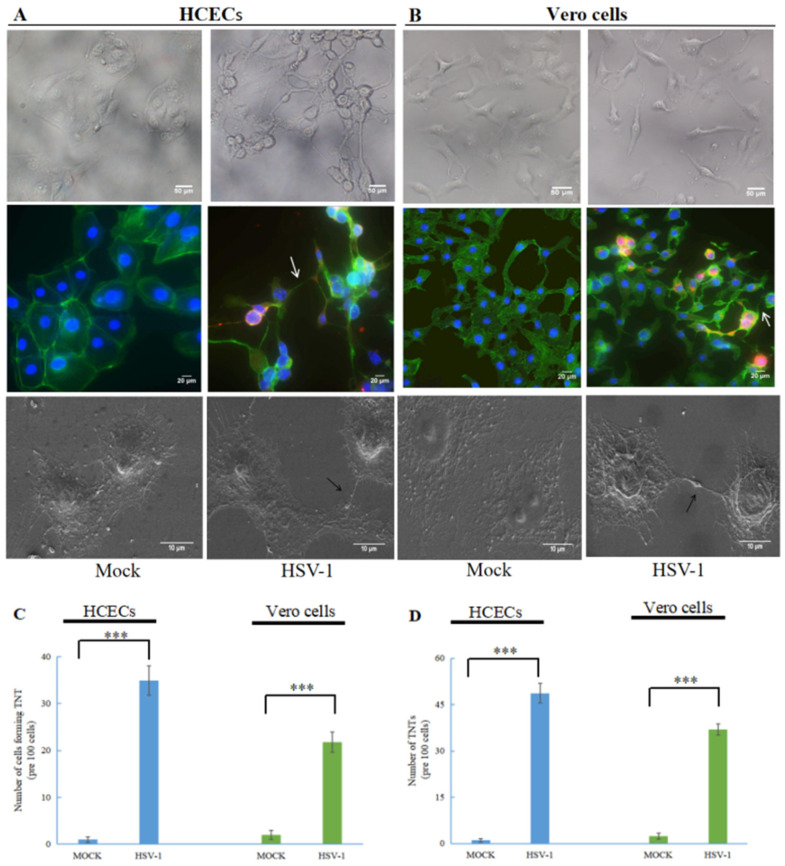
HSV-1 induces the formation of tunneling nanotubes (TNTs) in human cornea epithelial cells (HCECs) and Vero cells. HCECs (**A**) and Vero cells (**B**) at 24 h.p.i. with wild-type HSV-1 (multiplicity of infection (MOI) of 1) were photographed with an optical microscope, (200×); HCECs at 24 h.p.i. with wild-type HSV-1 (MOI of 1) were paraformaldehyde-fixed, permeabilized and stained for F-actin (FITC-Phalloidin, green staining), and HSV-1 proteins were detected using anti-HSV-1 gD antibody (red staining). White arrows indicate TNTs (400×); HCECs at 24 h.p.i. with wild-type HSV-1 (MOI of 1) were fixed with glutaraldehyde and observed using scanning electron microscopy, and black arrows indicate TNTs (2000×); graphs showing the number of TNTs (**C**) and the number of cells forming TNT (**D**) between infected cells (means ± SD) of triplicate assays (***, *p* < 0.001).

To analyze the effect of HSV-1 on TNT formation, we infected HCECs and Vero cells with wild-type HSV-1. As shown in Figure 1, compared to uninfected cells (mock), the morphology and intercellular connection of HCECs (Figure 1A) and Vero cells (Figure 1B) after HSV-1 infection changed. The morphology of HCECs and Vero cells were transformed under an optical microscope. In HCECs, especially, uninfected epithelial cells are polygonal, but after HSV-1 infection the polygonal shape of the HCECs basically disappeared. Using an immunofluorescence microscope and scanning electron microscope (SEM), we observed TNT-like connections between the two treatment cells after HSV-1 infection. To confirm whether the observed connections were TNTs, we used an actin-tracker to label F-actin (FITC- Phalloidin, green staining) and the presence of TNTs was confirmed at 24 h after HSV-1 infecting. The above results indicated that HSV-1 could stimulate the formation of TNTs in HCECs and Vero cells. 

We further quantitatively analyzed the formation of TNTs in HCECs and Vero cells stimulated by HSV-1 (Figure 1C), and the number of cells connected via TNTs was also quantified. For uninfected HCECs and Vero cells, TNTs could hardly be observed (<5%). After HSV-1 infection, a significant increase was found in both the cells forming TNTs and the number of TNTs. Around 30–40% of HCECs and 20–30% of Vero cells formed TNTs after HSV-1 infection (Figure 1D). 

### 3.2. TNTs Are Involved in HSV-1 Transmission

**Figure 2:**
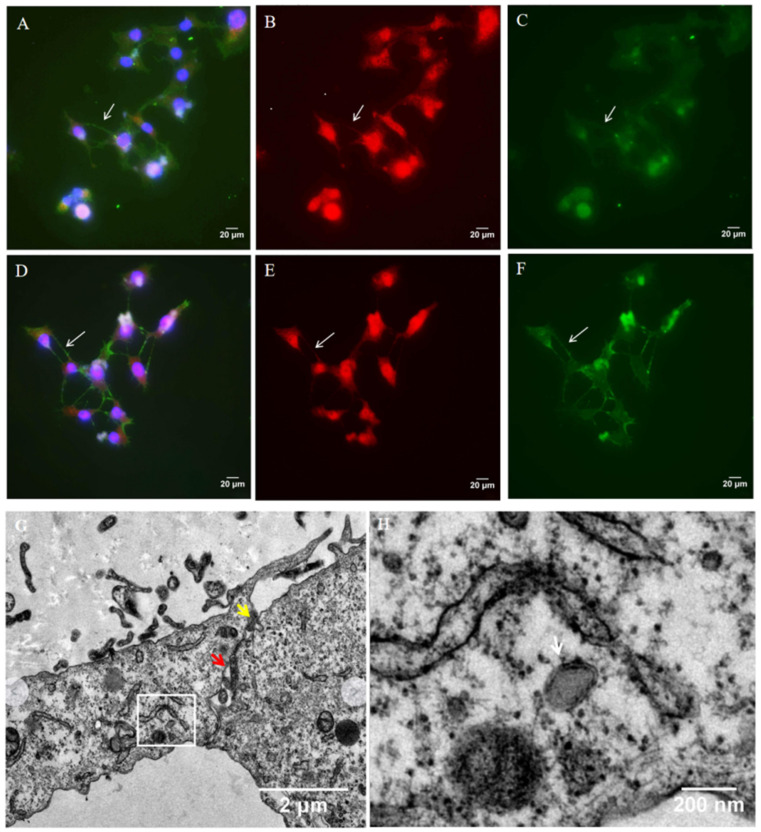
Detection of HSV-1 in tunneling nanotubes. Vero cells were infected with wild-type HSV-1 (multiplicity of infection (MOI) of 1). At 24 h.p.i., the cells were fixed and stained. HSV-1 proteins were detected using anti-HSV-1 gD (**A**,**B**) and gE (**D**,**E**) antibody (red staining). (**B**,**E**) in the absence of F-actin staining. (**C**,**F**) in the absence of gD or gE antibody staining. (**A**,**C**) The cells were also stained for F-actin (green staining) and nuclei (blue staining) (400×). Images were observed using fluorescence microscope; white arrows indicate viral proteins present in TNTs. (**G**,**H**) HCECs were cultured on 3D collagen gel with wild-type HSV-1 (MOI of 1); at 24 h.p.i., the cells were fixed and observed with transmission electron microscopy (TEM). (**G**) Yellow arrow indicates cytoplasmic fusion, and red arrow indicates loose area with gap between two cells. (**H**) White arrow indicates virion presents in TNTs; (**H**) is an enlarged view of the white box in (**G**).

TNTs are described as open-ended connections allowing the passage of cellular cargoes. Thus, we performed immunofluoresence staining to determine whether HSV-1 can be transported through TNTs. Vero cells were infected with wild-type HSV-1. At 24 h.p.i., the cells were fixed and analyzed for the presence of virus protein with anti-HSV-1 gD (Figure 2A,B, red staining) and gE (Figure 2D,E, red staining). F-actin was previously shown to be present in all types of TNTs, so the cells were also stained for F-actin (Figure 2A,C–E, phalloidin- Alexa Fluor 488, green staining). For Vero cells, HSV-1 gD and gE were detected in TNTs (Figure 2B,E). Almost all TNTs contained HSV-1 gD or gE protein, which suggested that HSV-1 could be transported through TNTs. 

The TEM imaging of HSV-1-infected HCECs cultured on three-dimensional (3D) collagen gel showed that virion was present in TNT (Figure 2H). The contact zone between a TNT and acceptor cell in HCECs was also detected using TEM. In the contact zone, we observed the cytoplasmic fusion area and the loose area with gaps between the two cells (Figure 2G). This phenomenon suggests that HSV-1 likely transfers directly from TNT to the receptor cell via cytoplasmic fusion. 

### 3.3. CK666 Inhibits TNT Formation Induced by HSV-1 Infection

**Figure 3:**
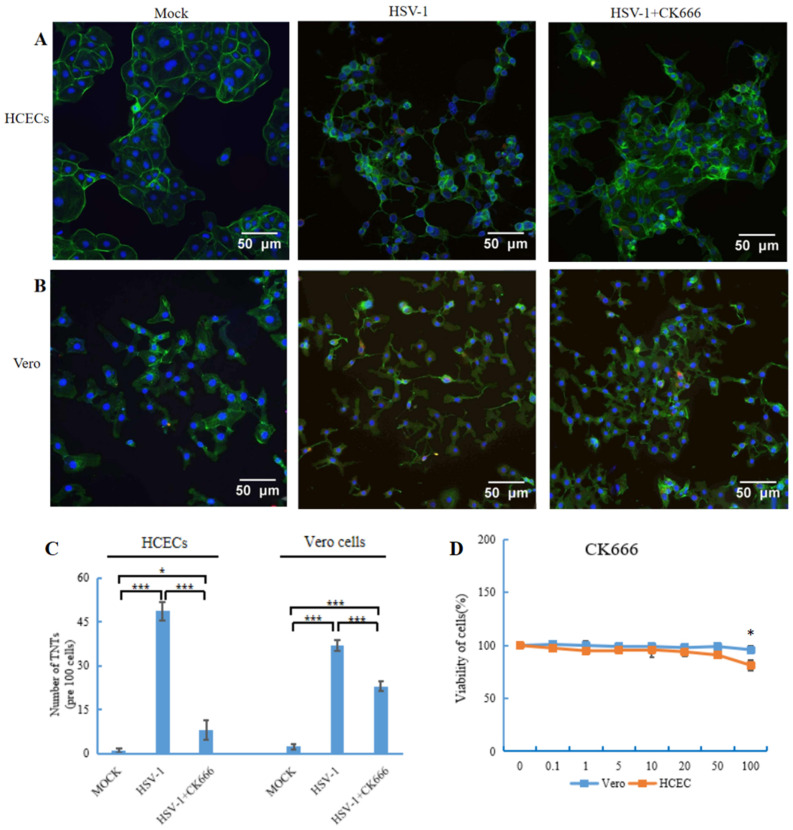
Fluorescence microscopy images showed the effect of CK666 on the formation of TNTs. (**A**,**B**) HCECs and Vero cells were stained with phalloidin green to visualize the nanotubes. Three images (per experiment) were taken per condition, with −100 cells examined, and the number of TNTs was counted. (**C**) Graph shows a significant reduction in the number of TNTs in the presence of CK666 (50 μM). Data are presented as mean ± SD. Student’s *t*-test was performed to compare between the groups, and the following significance values were considered: * *p* < 0.05, *** *p* < 0.001. (**D**) Cell viability of human corneal epithelial cells and Vero cells in the presence of CK666. Cells were treated with different concentrations of inhibitors, 0.1–100 μM, for 24 h, and the cell viability was measured by MTT assays. Only 100 μM was found to be toxic in two types of cells. Data shown are of three replicates.

The Arp2/3 complex plays an important role in actin cytoskeletal remodeling, but the role of the Arp2/3 complex in TNT formation is still unclear. To evaluate the role of the Arp2/3 complex in TNT formation, molecule compound CK666 (AdooQ, Irvine, CA, USA) was used to inhibit the activity of the Arp2/3 complex. A permissible concentration of CK666 was selected by using a cell viability assay. The effect of CK666 on the viability of HCECs and Vero cells was detected with MTT assays. CK666 showed no toxicity until a concentration of 100 μM (Figure 3D), so 50 μM CK666 was applied to the HCECs and Vero cells. The results showed that HSV-1 could still infect the Vero cell when CK666 was used to pre-treat theVero cell (Supplementary Figure S1). 

We used immunofluorescence to detect the expressions of gD and gE proteins that may play different roles in the HSV-1 replication cycle. The results showed that after Vero cells were infected with HSV-1 and then treated with CK666 for 4 h, 8 h, 12 h and 24 h, the expressions of gD and gE proteins could still be detected in cells and TNTs. These results indicate that after CK666 treatment, Vero cells can still be infected by HSV-1 and HSV-1 can still replicate in Vero cells (Supplementary Figure S2).

The HCECs and Vero cells were infected by HSV-1 for 1 h, then changed to the medium containing CK666 (50 μM). After 24 h incubation, cells were fixed, stained with phalloidin (green staining) and DAPI (blue staining), and then imaged using a fluorescence microscope. The results suggested a visible reduction of TNTs in both HCECs (Figure 3A) and Vero cells (Figure 3B) after CK666 treatment. We further counted the number of TNTs, and found that TNTs decreased significantly in two types of cells, especially in HCECs, by nearly 80%. The number of TNTs between Vero cells decreased by 40% (Figure 3C).

### 3.4. CK666 Can Weaken Intercellular HSV-1 Spread 

**Figure 4:**
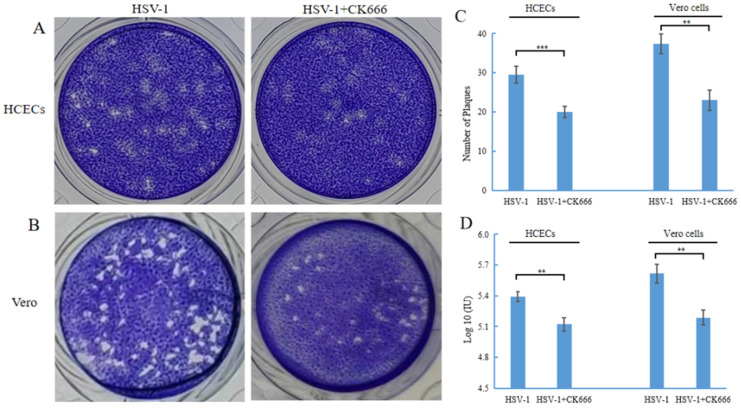
CK666 affects the transmission of HSV-1. Confluent monolayers of cell plates in 24-well culture were infected with HSV-1 for 1 h, then treated with/without 50 μM CK666 for 24 h in HCECs (**A**) and Vero cells (**B**) and processed for the plaque assay. (**C**) Graph shows a reduction in the number of plaques in HCECs and Vero cells treated with CK666 compared to control cells. (**D**) HCECs and Veros were infected with wild-type HSV-1 (MOI of 1), and the effect of the addition of 50 μM CK666 on the HSV-1 DNA level in HCECs and Vero cells was quantified with qPCR. Data represent mean ± SD of triplicate assays. Student’s *t*-test was used to compare between the groups. ** *p* < 0.01, *** *p* < 0.001.

The gD of HSV-1 found in TNT indicates that HSV-1 can be spread through TNT. CK666 can inhibit the formation of TNT and may inhibit the intercellular transmission of HSV-1. We performed a plaque assay to compare the number of plaques in HSV-1 infected cells with or without CK666. The HSV-1 plaque-forming unit reflected both virus yield per cell and the ability of the infection to spread from cell to cell. The results showed that the number of plaques after CK666 treatment was significantly reduced compared with those without CK666 treatment (Figure 4A,B). After HCECs were infected with HSV-1, the level was 2 × 10^5^ PFU/mL; after treating with of CK666, it was 1.3 × 10^5^ PFU/mL. After Vero cells were infected with HSV-1, it was 4.4 × 10^5^ PFU/mL; after treatment with of CK666, it was 2.2 × 10^5^ PFU/mL. In other words, CK666 effectively reduced the intercellular transmission of HSV-1 in HCECs and Vero cells. 

The effect of CK666 on the DNA expression of HSV-1 in HCECs and Vero cells was also detected. HCECs and Vero cells were infected with HSV-1 for 1 h, then changed to the medium containing CK666 (50 μM), and incubated for 24 h, and the DNA levels of HSV-1 in HCECs and Vero cells were detected after CK666 treatment. The DNA levels of HSV-1 in HCECs and Vero cells were significantly decreased (Figure 4D). Combined with the above results, CK666 can weaken the intercellular transmission of HSV-1. 

## 4. Discussion

Herpes viruses use multiple strategies for transmission. HSV-1 spread usually occurs via two major routes; cell–cell spread and cell-free release [16]. For the nonadjacent intercellular transmission of viruses, previous studies have believed that the virions were released from infected cells into the extracellular environment via exocytosis, then adsorbed to uninfected cells, and membrane fusion occurred, and invaded into the cells, which is the so-called “cell free release” route [17,18]. Currently, some types of viruses have been revealed to be able to induce nanotube formation between nonadjacent cells and spread via nanotubes [19,20,21,22]. Thus, cell-free release may not be the only mode of virus transmission between non-adjacent, long-distance cells. In this study, we demonstrated that HSV-1 can induce the formation of tunneling nanotubes between HCECs, and also between Vero cells. We also detected the presence of fluorescent-labeled HSV-1 glycoprotein D (gD) and gE in the nanotube. Also, the presence of virion in tunneling nanotubes was observed through TEM. These results confirmed that HSV-1 can spread between cells via TNTs. 

We observed the membrane fusion between TNT and receptor cells. After TNT makes contact with receptor cells, the membranes fuse to form a channel for HSV-1 transport. This is a conductive way for HSV-1 to spread through TNTs. HSV-1 can transmit through TNT to avoid the barriers of the multistep process (such as fusion, entry, release by exocytosis) and enhance viral spread. In addition, the virions in nanotubes can be protected from the host immune response. The virions hidden in TNT are not easily attacked by the host’s immune system [23,24]. Here, we provide evidence for HSV-1’s intercellular spread through TNTs in HCECs and Vero cells. Thus, the transmission of HSV-1 in cells is not limited to cell-free release spread and cell-to-cell intimate contact [25].

TNTs have three-dimensional (3D) structures. Some works in the literature have reported that cancer cells can construct TNT projections in 3D hydrogel to directly contact distant cells [26]. However, almost all studies used two-dimensional cell culture models to study the formation of virus-induced TNT. Whether HSV-1 can induce TNT formation in 3D gel is unknown. Type I collagen was used to simulate the 3D environment of the corneal stroma. In this study, we simulated the microenvironment of 3D corneal tissue to observe the intercellular ultrastructure of HSV-1-infected HCECs on the 3D gel, which was composed of type I collagen. TNTs could also be formed between HCECs growing in the 3D gel. HSV-1 can induce TNT formation in 3D collagen gel, which opens the possibility of exploring the formation of TNT in vivo. Therefore, we presume that the cells in human cornea tissue may also form TNTs even though they are wrapped by collagen; further research is needed to confirm this. Several studies have shown that different types of cells could form TNTs [27,28]. TNT is formed between HCECs grown on type I collagen gel, which provides a research idea for studying the formation of TNT between HCECs and keratocyte, as well as between keratocyte and endothelial cells and proposing a hypothesis of HSV-1 spread in the cornea tissue through TNT.

From the above results, we demonstrated that HSV-1 can transmit through TNTs. Inhibiting the formation of TNTs will become a possible way to inhibit HSV-1 spread. At present, the mechanistic study of TNT formation has not been clearly reported in virus transmission. Some studies have shown that TNTs are tubular, based on F-actin [29,30,31,32,33], and the Arp2/3 complex is a potential target for investigating the mechanisms of TNT formation. In this study, we used a small molecule compound, CK666, to bind the subunits of the Arp2/3 complex, so as to prevent the conformational rearrangement of these subunits required for Arp2/3 complex activation [34]. Our results showed that the number of TNTs between HCECs and between Vero cells decreased significantly after CK666 intervention. These data indicated that the Arp2/3 complex is involved in the TNTs’ conformation, and that CK666 could reduce the formation of TNTs.

The direct connection between HCECs or Vero cells through TNTs provides a convenient channel for HSV-1 transmission. We observed that, in the presence of CK666, the amount of plaque and the DNA level of HSV-1 in HCECs and Vero cells were significantly decreased (Figure 4). Therefore, TNTs could mediate the spread of HSV-1 between HCECs or Vero cells, and in the presence of CK666 the HSV-1 transfer between HCECs or Vero cells was weakened. It is suggested that CK666 may weaken the spread of HSV-1 by affecting the formation of TNTs, or weaken the spread of viruses by affecting the inner cytoskeleton dynamics. While further research is needed in the future, these results indicate that CK666 and its analogues may become potential medicines for the treatment of HSV-1. 

## 5. Conclusions

In summary, we confirmed that TNT is a new spreading mode of HSV-1. HSV-1 could induce TNTs’ formation, and spread via TNTs. CK666, an inhibitor of the Arp2/3 complex, was proved to inhibit the formation of TNT, limiting the spread of HSV-1. Therefore, CK666 may become a potential lead compound to slow the spread of HSV-1, providing a new approach for the treatment of HSV-1. This study has enriched the knowledge of methods of HSV-1 transmission, and proposed the view that HSV-1 can be transported between distant cells by forming microtubules. For our future studies, we will focus on whether TNTs can be formed between different cell types, and whether HSV-1 can be transmitted between different cell types through TNTs.

## Data Availability

All relevant datasets generated in this study are included in the manuscript.

## Supplementary Materials

The following supporting information can be downloaded at: https://www.mdpi.com/article/10.3390/microorganisms11081916/s1. Figure S1. HSV-1 gD and gE protein was detected after CK666 pretreatment of Vero cells, Figure S2. After CK666 intervened Vero cell for 4 h, 8 h, 12 h and 24 h, HSV-1 gD and gE proteins were detected in Vero cell.
