# Supplementary material for: Herpes Simplex Virus Type 1 Infection Induces the Formation of Tunneling Nanotubes
Source: Microorganisms. 2023 Jul 28;11(8):1916. doi: 10.3390/microorganisms11081916 (PMC10456791; doi:10.3390/microorganisms11081916)
Supplement: Supplementary file 1 [file microorganisms-11-01916-s001.zip › microorganisms-2435893-supplementary.pdf]

microorganisms

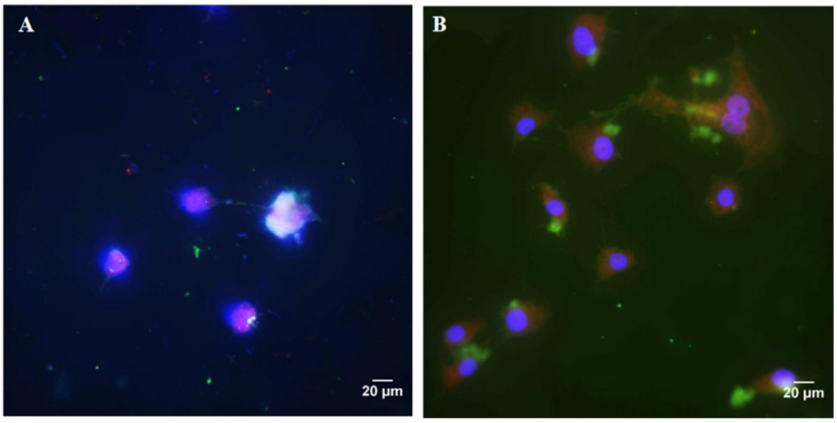

Supplementary Figure S1. HSV-1 gD and gE protein was detected in Vero cells. 50μM CK666 to interfere with Vero cells for 12 hours in advance, and then detected the expression of gD and gE in cells 24 h.p.i. with wild-type HSV-1 (MOI of 1) (DMEM medium containing 50M CK666 was still used to culture cells during HSV-1 infection of Vero cells). The cells were also stained for anti-HSV-1 gD (A) and gE(B) antibody (red staining), F-actin (green staining) and nuclei (blue staining) (400×)

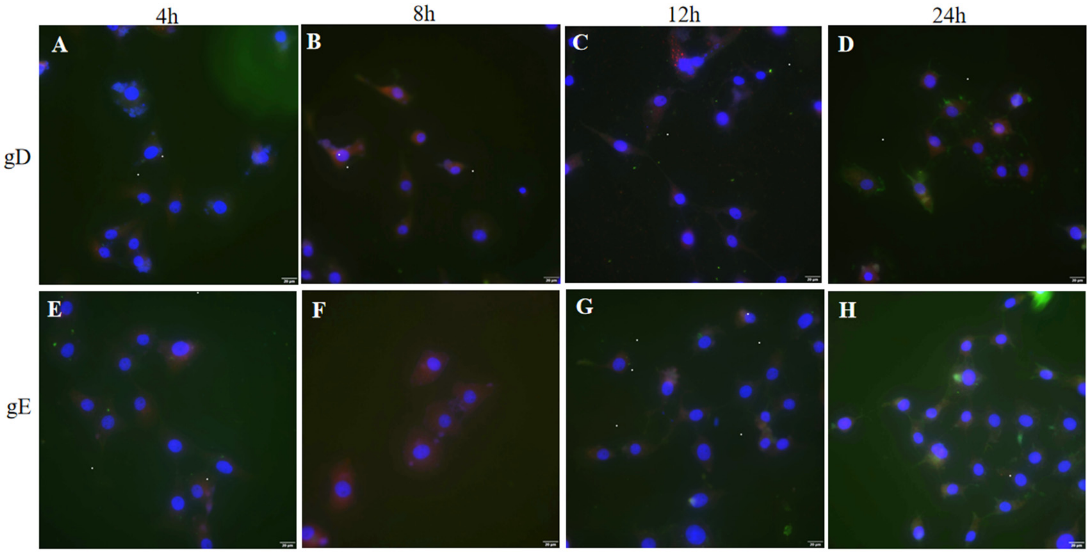

Supplementary Figure S2. HSV-1 gD and gE protein was detected in Vero cells. Expression of gD and gE in Vero cell after HSV-1(MOI of 1) infection combined with CK666 intervention for 4h, 8h, 12h and 24h.The cells were also stained for anti-HSV-1 gD (A,B,C,D) and gE(E,F,G,H) antibody (red staining), F-actin (green staining) and nuclei (blue staining) (400×)
